# ABC classification of posterior shoulder instability

**DOI:** 10.1007/s11678-017-0404-6

**Published:** 2017-04-20

**Authors:** Philipp Moroder, Markus Scheibel

**Affiliations:** Department of Shoulder and Elbow Surgery, Center for Musculoskeletal Surgery, Charité – Universitaetsmedizin Berlin, Augustenburger Platz 1, 13353 Berlin, Germany

**Keywords:** ABC Classification, Posterior shoulder instability, Functional instability, Posterior subluxation, Locked posterior dislocation, ABC Klassifikation, Hintere Schulterinstabilität, Funktionelle Instabilität, Hintere Subluxation, Verhakte hintere Luxation

## Abstract

Posterior glenohumeral instability (PGHI) is a commonly under- and misdiagnosed pathology owing to its variety of clinical presentations. In order to facilitate diagnosis and treatment, the simple yet comprehensive ABC classification for PGHI is based on the underlying pathomechanical principles and current standard of treatment. Three main groups of PGHI are distinguished based on the type of instability: A (first time), B (dynamic), C (static). Two subtypes further differentiate these groups in terms of their specific pathomechanism and provide a guideline in the choice of appropriate treatment: A (1, subluxation; 2, dislocation), B (1, functional; 2, structural), C (1, constitutional; 2, acquired). While conservative treatment is warranted in most patients with type 1 PGHI (A1, B1, C1), surgical treatment should be considered on an individual basis in patients with type 2 PGHI (A2, B2, C2), while keeping in mind that the different groups and subtypes can overlap, co-exist, or even progress from one to another over time. Of course the necessity for surgical treatment depends on the extent of the structural defects, on the severity of symptoms, on the chronicity, as well as on patient-specific functional demand, age, and health status. Nonetheless, the ABC classification helps to correctly diagnose the type of PGHI and provides a guideline for the generally recommended type of treatment.

**Electronic supplementary material:**

The online version of this article (doi: 10.1007/s11678-017-0404-6) contains supplementary material (Video), which is available to authorized users.

## Zusammenfassung

Die posteriore glenohumerale Instabilität (PGHI) wird aufgrund der vielfältigen klinischen Manifestationen häufig nicht erkannt oder fehldiagnostiziert. Um die Diagnose und Therapiefindung zu erleichtern, wurde die vereinfachende, jedoch umfassende ABC-Klassifikation für PGHI unter Berücksichtigung der zugrunde liegenden pathomechanischen Prinzipien sowie der gegenwärtigen Behandlungsstandards geschaffen. Drei Hauptgruppen werden aufgrund der Art der Instabilität unterteilt: A (erstmalig), B (dynamisch), C (statisch). Zwei Subtypen unterteilen die 3 Hauptgruppen auf Basis der spezifischen pathomechanischen Ursache der Instabilität noch weiter und stellen eine Richtlinie für die Therapiefindung dar: A (1: Subluxation; 2: Dislokation), B (1: funktionell; 2: strukturell), C (1: konstitutionell; 2: erworben). Während ein konservativer Therapieversuch bei den meisten Patienten mit einer PGHI vom Typ 1 empfehlenswert ist, sollte bei Patienten mit einer PGHI vom Typ 2 eine chirurgische Intervention auf individueller Basis erwogen werden, wobei zu bedenken ist, dass die verschiedenen Gruppen und Subtypen sich überlappen, koexistieren oder im Verlauf der Zeit ineinander übergehen können. Dabei hängt die Notwendigkeit einer chirurgischen Intervention vom Ausmaß der strukturellen Schäden, der Schwere der Symptome, der Chronizität sowie dem funktionellen Anspruch, Alter und allgemeinen Gesundheitszustand des Patienten ab. Dennoch stellt die ABC-Klassifikation ein Hilfsmittel zur korrekten Diagnose- und Therapiefindung bei PGHI dar.

Posterior glenohumeral instability (PGHI) is less frequently encountered than anterior glenohumeral instability (AGHI). Nonetheless, a recent study suggests that PGHI accounts for up to 24% of all young and active patients surgically treated for shoulder instability, which is much higher than the 5% prevalence often mentioned in the literature [33].

A reason for this large discrepancy might be that PGHI is one of the most commonly misdiagnosed or not-recognized shoulder pathologies. In general, the clinical symptoms associated with PGHI are often much more subtle than the symptoms encountered in AGHI [20, 29]. While patients with AGHI most of the time are able to point out their problem themselves, PGHI patients are not always aware of their shoulder joint dislocating or subluxating posteriorly and instead often report unspecific shoulder discomfort, pain, or functional deficits. Similarly, the clinical diagnosis of PGHI is more challenging, since patients often lack the typical feeling of apprehension in provocative arm positions, which, by contrast, clearly pinpoint the problem in AGHI patients. PGHI patients sometimes only complain about minor functional impairment and moderate levels of pain during high-demand activities. Even in the presence of a chronic locked posterior dislocation, the residual shoulder function is surprisingly good sometimes with preserved elevation above 90° and limited pain.

Another difficulty in the diagnosis and treatment of PGHI is the multifaceted nature of this pathology, which poses a challenge to the treating physician [29].

In order to facilitate diagnosis and improve treatment of PGHI, it is necessary to find a common nomenclature in terms of a simple yet comprehensive classification.

## The ABC classification

**Fig. 1:**
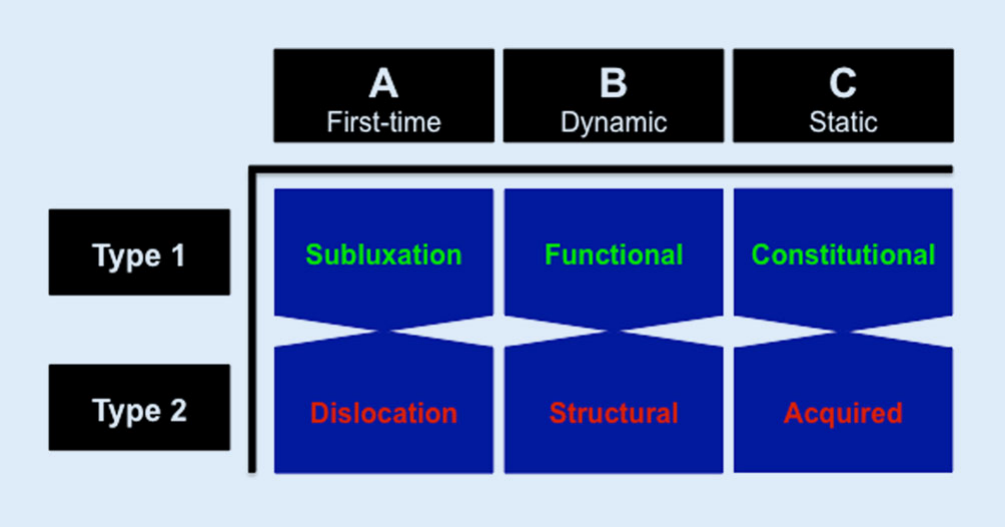
The ABC classification of posterior shoulder instability

Since clinical classifications should not only fulfill an academic purpose but should contain guiding principles on the necessary ensuing treatment [1], the ABC classification distinguishes three groups of PGHI with two different subtypes based on the pathomechanical type of instability and the current standard of treatment (Fig. 1).

### Group A

Group A includes all patients with an acute first-time posterior instability event that can either have occurred in terms of a subluxation without engagement of the humeral head with the posterior glenoid rim (A1) or in terms of a dislocation with temporary or persisting engagement (A2). Distinction of this group and the two subtypes is possible by a combination of taking the patient’s history and imaging studies, both applicable even in the acute and painful setting.

#### A1: Acute posterior subluxation (Fig. 2)

Pathomechanism: The humeral head translates posteriorly barely over the posterior glenoid rim and spontaneously returns into a reduced position usually without causing major bony or soft-tissue defects.

**Fig. 2:**
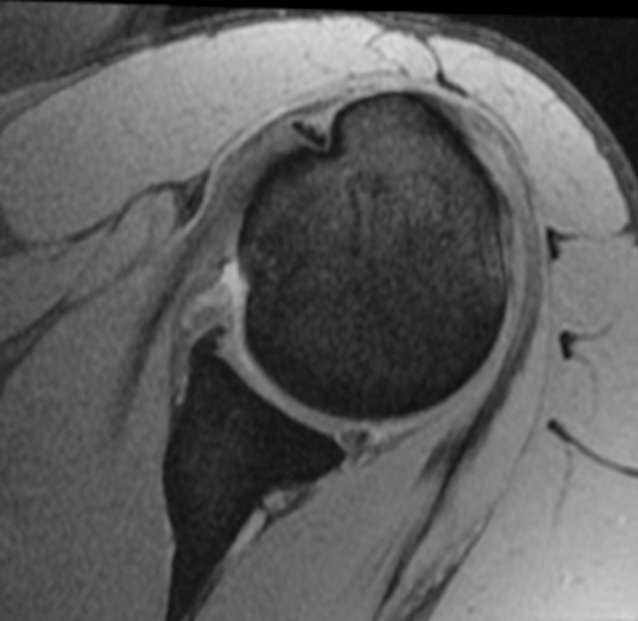
First-time posterior subluxation (A1). Magnetic resonance image of a young male patient who sustained an acute posterior shoulder subluxation with small reverse Hill–Sachs lesion and minor posterior capsulolabral damage during sports participation without major trauma

Cause: This type of PGHI is mostly caused by a minor or moderate mechanical trauma with the arm in forward flexion and internal rotation or sometimes even merely by an inadvertent movement of the arm.

Clinical presentation: Patients usually complain of moderate pain exacerbated by forced internal rotation along with slight limitation of range of motion that quickly improves over time.

Imaging:

1. Standard radiographs confirm joint reduction (true anteroposterior (AP), Y‑view, Velpeau view, or if obtainable axillary view) and may show signs of minor bony humeral (reverse Hill–Sachs lesion) or glenoid defects (posterior bony Bankart).
2. Magnetic resonance imaging (MRI) is the imaging modality of choice as it helps to identify capsulolabral complex lesions, cartilage damage, tendon tears, and also reverse Hill–Sachs lesions that are typically highlighted by a bone marrow edema.
3. Computed tomography (CT) imaging allows for the exact analysis of humeral head and especially glenoid integrity in the case of suspected bony defects.

Treatment:

1. In the case of no significant bony or soft-tissue defects, conservative treatment is warranted.
2. Immobilization in internal rotation has been described to improve posterior labrum reduction after a sustained posterior capsulolabral detachment [6]. Only in young patients with high functional demand should early surgical reconstruction of a diagnosed posterior capsulolabral tear be considered.
3. Critical humeral or glenoid defects necessitating surgical treatment are seldom encountered in this group of PGHI [24].

#### A2: Acute posterior dislocation (Fig. 3)

Pathomechanism: The humeral head dislocates posteriorly sustaining an impaction fracture of the anteromedial surface of the humeral head (reverse Hill–Sachs lesion) that temporarily or permanently remains engaged with the posterior glenoid rim (locked situation).

**Fig. 3:**
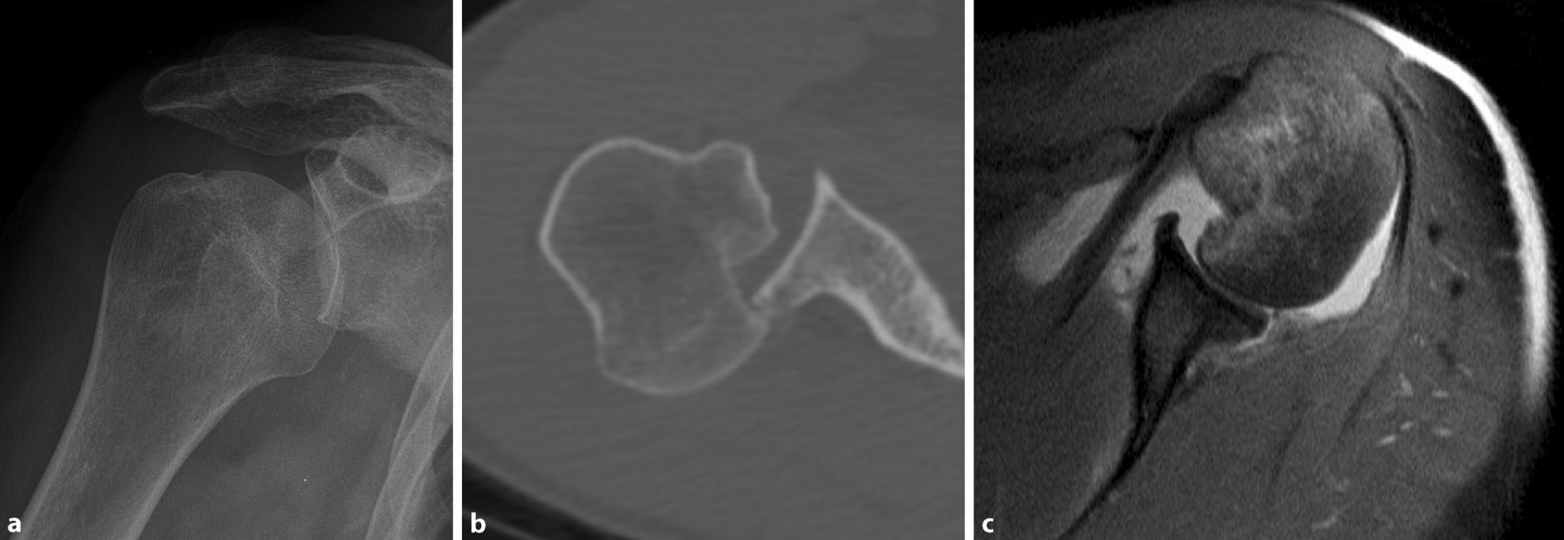
First-time posterior dislocation (A2) X‑ray (**a**) and computed tomography image (**b**) of an acute locked posterior shoulder dislocation with large reverse Hill–Sachs defect as well as magnetic resonance scan (**c**) of a reduced acute posterior shoulder dislocation with large Hill–Sachs defect and posterior Bankart lesion

Cause: Mechanical trauma with the arm in forward flexion and internal rotation or pathological muscle contractions during seizures or electrical accidents are the most common cause.

Clinical presentation: Patients typically present with acute pain exacerbated by motion in general. In the case of a locked dislocation, changes in the shoulder contour with prominent coracoid tip are sometimes visible and a blocked external rotation can be observed while internal rotation and elevation are painful but frequently partially preserved.

Imaging:

1. Standard radiographs depict locked situations (true AP, Y‑view, Velpeau view, or if obtainable axillary view) and may show traces of bony humeral (reverse Hill–Sachs lesion) or glenoid defects (posterior bony Bankart)
2. CT imaging ensures the exact analysis of bony humeral and glenoid defects.
3. MRI is useful for the evaluation of the capsulolabral complex, cartilage, tendons and also reverse Hill–Sachs lesions that are typically highlighted by a bone marrow edema.

Treatment:

1. In the case of a locked dislocation, either closed or open reduction depending on the presence of concomitant proximal humeral fractures and the time passed since dislocation is warranted.
2. Reverse Hill–Sachs defects are a major risk factor for recurrence of instability [30]. If the gamma angle of the defect exceeds 90° [23], consider acute surgery in terms of defect disimpaction (within 2 weeks), bone grafting, soft tissue coverage, or arthroplasty (in older patients).
3. Posterior glenoid rim fractures warrant early surgical treatment in terms of an indirect suture anchor repair (small fragment) or direct screw fixation (large fragment) in young patients in order to prevent resorption of medialized fragments and subsequent glenoid bone deficiency potentially leading to recurrence of instability as described for AGHI [26]. In older patients the repair of large defects should be considered.
4. Only in young patients with high functional demand should early surgical reconstruction of a diagnosed posterior capsulolabral tear be considered. Otherwise, immobilization in internal rotation to improve posterior labrum reduction is recommended [6].
5. Conservative treatment is generally possible if no critical bony or soft-tissue defect is discovered.

### Group B

Group B includes all patients with recurrent dynamic posterior instability events that occur during motion either in form of a functional instability (B1) or a structural instability (B2). While patient history helps to identify this group of PGHI, clinical examination and imaging studies are the method of choice to differentiate between the two subtypes.

#### B1: Functional dynamic posterior instability (Video 1)

Pathomechanism: Pathological activation pattern of the rotator cuff muscles as well as periscapular musculature leads to mostly posterior [34] dislocation of the humeral head during movement of the arm usually without creating structural damage. This type of PGHI is often associated with structural deficiencies such as hyperlaxity, posterior capsular redundancy, flattened glenoid concavity, or increased glenoid retroversion due to glenoid dysplasia.

Cause: Typically caused by atraumatic development of aberrant shoulder muscle activation pattern during adolescence.

Clinical presentation: Painful or painless involuntary dislocation of the humeral head during movement of the arm often accompanied by a pathological movement pattern of the scapula can be observed. Sometimes voluntary dislocations can be provoked by the patients as well. Instability mediated by pathological muscle activation patterns can be further differentiated and temporarily overcome by use of the resisted external rotation test/wall-slide test[11, 16] (hypoactive external rotators) as well as the scapular assistance test [4, 18] (scapular dyskinesis). Hyperlaxity should be evaluated by means of the Beighton score [2], Drawer test [10], sulcus sign [27], and Gagey test [8].

Imaging:

1. While this group of PGHI is best identified with a clinical examination, MRI should additionally be employed to ensure the absence of acquired structural defects or constitutional deficiencies.

Treatment:

1. Conservative treatment with intensive physiotherapy to normalize muscle activation pattern is recommended [34]. The physiotherapy should be focused on scapular motion coordination and activation of external rotators [16, 19].
2. A pilot study using a so-called shoulder pacemaker showed excellent preliminary results [25].
3. Surgical treatment is not warranted in this group of patients as it usually fails to restore stability and often results in pain as well as limited function [14, 15, 21, 31]. Only if the posterior instability persists despite successful treatment of the functional component might surgical treatment of evident structural deficiencies be attempted as a salvage procedure [7].

#### B2: Structural dynamic posterior instability (Fig. 4)

Pathomechanism: Structural damage including posterior Bankart lesions, capsular insufficiency due to repetitive microtrauma, posterior glenoid bone loss, or critical reverse Hill–Sachs lesions cause recurrent PGHI during axial loading of the flexed and internally rotated arm. This type of instability can be enhanced by a multifactorial combination of individual constitutional structural and functional deficiencies including hyperlaxity, capsular redundancy, flattened glenoid concavity, increased glenoid retroversion due to glenoid dysplasia, or scapular dyskinesis.

**Fig. 4:**
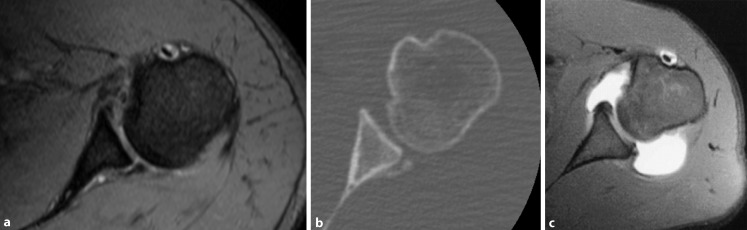
Structural dynamic posterior instability (B2). Dynamic structural posterior shoulder instability with posterior bony Bankart lesion visible on magnetic resonance (MR) (**a**) and computed tomography (**b**) images. MR arthrography of a patient with posterior capsular redundancy and posterior labral damage as an example of combined structural deficiency and structural damage (**c**)

Cause: A single mechanical trauma or involuntary muscle contraction due to a seizure or electrical accident as well as repetitive microtrauma can be the cause of structural damage and lead to this type of PGHI. In the presence of constitutional structural deficiencies, minor trauma and structural damage can suffice to cause dynamic PGHI.

Clinical presentation: Recurrent dislocations or subluxations occur during movements in flexion and internal rotation. However, they are often not recognized as instability episodes but rather perceived as pain, weakness, or clicking noise by the patients. Functional tests including the Jerk test [13], Kim test [20], as well as the load-and-shift test [13] are helpful for detecting this type of PGHI. Hyperlaxity should additionally be evaluated by means of the aforementioned clinical tests.

Imaging:

1. MRI is used for the evaluation of the posterior capsulolabral complex, cartilage, glenoid morphology, tendon tears, and to a lesser degree the extent of bony defects.
2. CT imaging is of advantage in the evaluation of posterior glenoid bone loss, reverse Hill–Sachs defects, and glenoid morphology.

Treatment:

1.: In the case of a painful and functionally impairing structural dynamic PGHI, consider planned surgical treatment including focused repair of the structural bony or soft-tissue defects encountered as well as addressing constitutional structural deficiencies:
a.: Reverse Hill–Sachs defect: If the gamma angle of the defect exceeds 90°, prevention of re-engagement of the defect by means of bone grafting or soft-tissue coverage is recommended [23].
b.: Posterior bony glenoid defect: While the threshold value for critical posterior glenoid bone defects has yet to be determined, there is consensus to treat extensive bone loss by the use of bone grafting [5, 22].
c.: Posterior capsulolabral defects and insufficiency: Arthroscopic posterior capsulolabral repair provides significant improvements in terms of stability, pain, and function in patients with capsulolabral tears or insufficiency. Capsulolabral tears treated with suture anchor repair result in a better clinical outcome than mere posterior capsular insufficiency treated with anchorless capsulolabral plications [3].
2.: In the case of painless structural dynamic PGHI with an additional functional muscle patterning component, conservative treatment can be successful in restoring stability [19] and should be focused on scapula positioning and strengthening of external rotators [16].

### Group C

Group C includes all patients with chronic static PGHI caused by either constitutional structural deficiencies (C1) or acquired structural defects (C2). This group of PGHI is best identified and subclassified by a combination of patient history, clinical examination, and imaging studies.

#### C1: Constitutional static posterior instability (Fig. 5)

The cause and pathomechanism of this type of PGHI are still poorly understood [35]. A possible explanation is constitutional force imbalances and scapular malpositioning leading to eccentric contact of the joint partners and eventually progressive eccentric posterior glenoid wear. Another cause might be excessive glenoid retroversion or in more severe cases the malformation of a glenoid ossification center leading to glenoid hypoplasia and static posterior humeral head subluxation.

**Fig. 5:**
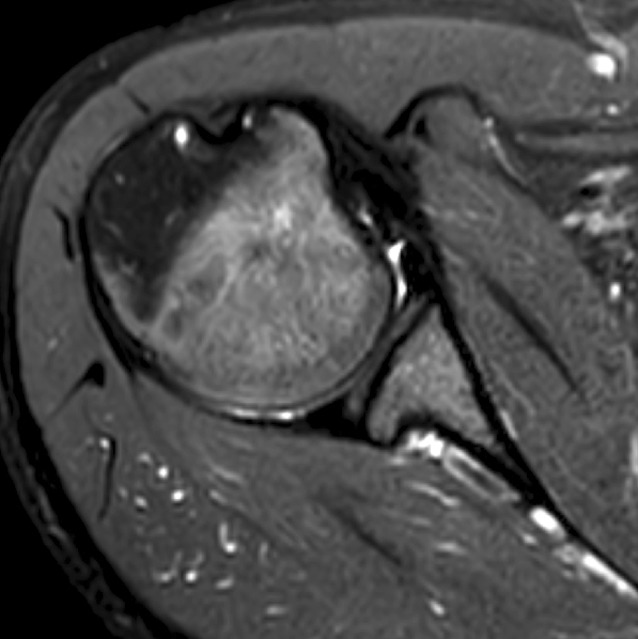
Constitutional static posterior instability (C1). Static constitutional posterior shoulder instability with increased posterior humeral head translation, congenital convex-shaped glenoid, and increased retroversion

Clinical presentation: This type of PGHI is mostly asymptomatic in early stages and therefore only detected as an incidental finding. Pain will only occur with increasing posterior cartilage damage due to progressive eccentric wear [35].

Imaging:

1. MRI is useful for the evaluation of static posterior humeral head translation, cartilage defects, labral damage, and degenerative changes with increased posterior glenoid wear.
2. CT scans allow one to analyze severe bony deformities in advanced stages of the pathology.

Treatment:

1. Conservative treatment including physiotherapy in an attempt to re-center the humeral head during early stages of the pathology or to reduce symptoms in the presence of advanced degenerative changes should be the first line of treatment. In order to reduce symptoms, shoulder-demanding sports and work activities should be avoided.
2. Surgical treatment options other than arthroplasty in adults with progressed degenerative changes include an anterior soft-tissue release combined with posterior capsulorrhaphy, posterior open-wedge glenoid osteotomy, and posterior bone block procedures, all of which have shown limited success [35].

#### C2: Acquired static posterior instability (Fig. 6)

Pathomechanism: Owing to an acquired structural damage mostly including large reverse Hill–Sachs lesions or posterior glenoid bone defects, the glenohumeral joint is permanently de-centered meaning that the humeral head remains in a posteriorly subluxated or dislocated position leading to degenerative changes over time. In children with brachial plexus birth palsy, the ensuing internal rotation contracture and rotational force couple imbalance can lead to static posterior humeral head subluxation and severe osseous developmental deficiencies including posterior glenoid rim dysplasia.

**Fig. 6:**
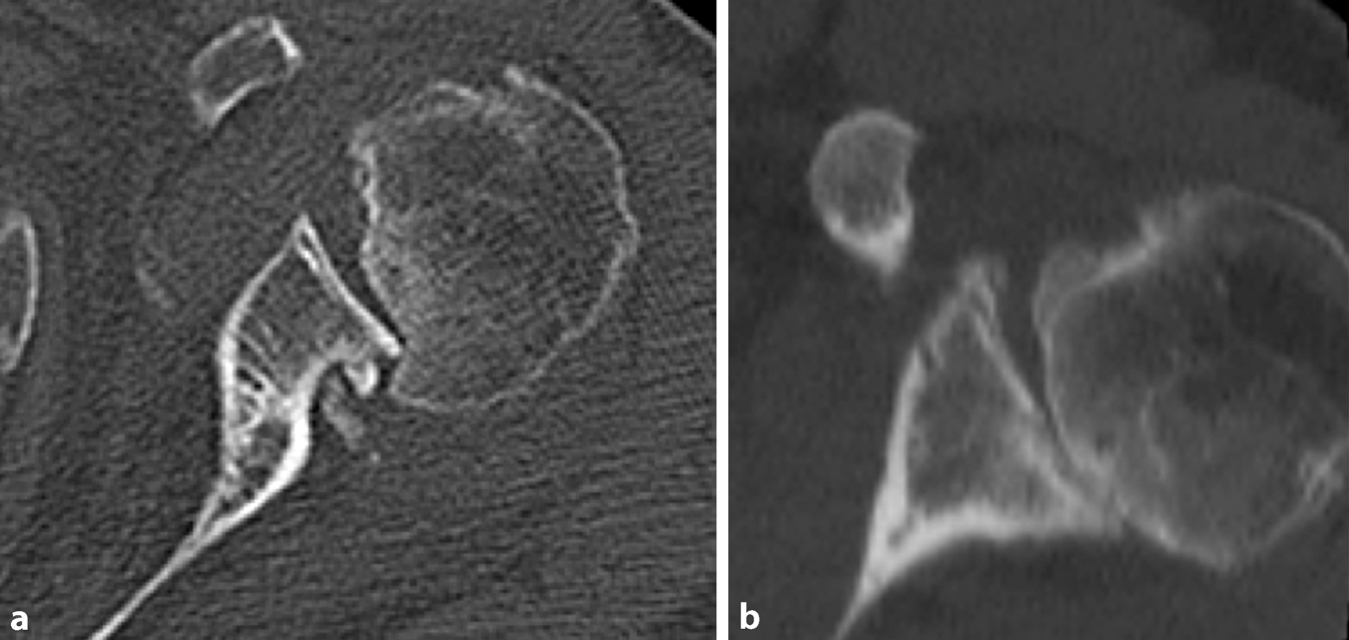
Acquired static posterior instability (C1). Static acquired posterior shoulder instability due to chronic locked posterior shoulder dislocation with large reverse Hill–Sachs defect (**a**) and due to untreated posterior glenoid rim impaction fracture with de-centered humeral head and progressed degenerative changes (**b**)

Cause: Mechanical trauma or pathological muscle contractions during a seizure or electrical accident are the main causes of this type of PGHI.

Clinical presentation: Depending on the degree of secondary osteoarthritis, a variable clinical presentation can be observed. Symptoms mostly include pain and limited motion especially concerning external rotation. Owing to excessive posterior humeral head translation, the shoulder contour is changed in some patients featuring a prominent coracoid tip. In the case of a chronic locked dislocation, external rotation is often severely reduced or even blocked while internal rotation and elevation are sometimes preserved to a certain extent.

Imaging:

1. Standard radiographs depict a posterior subluxation or locked dislocation (true AP and axillary view), show signs of bony humeral (reverse Hill–Sachs lesion), or glenoid defects and allow one to analyze the degree of secondary osteoarthritis.
2. CT imaging allows for exact evaluation of bony humeral and glenoid defects, posterior humeral head translation, as well as degenerative changes.
3. MRI helps to identify tendon tears, cartilage damage, and labral defects in addition to posterior humeral head translation and to a lesser degree the extent of bony defects

Treatment:

1.: Planned open reduction and surgical treatment including restoration of the articulating surfaces combined with soft-tissue balancing in order to avoid recurrence of posterior humeral head migration are recommended.
d.: Chronic locked reverse Hill–Sachs lesion: Bone grafting represents the joint-preserving treatment of choice [9] while rotational osteotomy remains a secondary option [17]. In cases of advanced osteoarthritis and advanced patient age, anatomical arthroplasty can be performed [36]; however, if soft-tissue balancing is not achievable in long-standing locked situations, a more constrained prosthesis design in terms of reverse shoulder arthroplasty must be considered.
e.: Posterior glenoid bone defects: Joint-preserving options include posterior bone block procedures [32] and posterior open-wedge glenoid osteotomy [28]. Depending on the degree of posterior glenoid bone loss and the achievability of soft-tissue balancing [12] either anatomical or reverse arthroplasty can be considered in older patients and cases of advanced osteoarthritis.
2.: Skillful neglect represents a treatment option in case where the patient is not cleared for surgery.

## Conclusion

The ABC classification offers a simple yet comprehensive classification of PGHI with clear distinction criteria and therapeutic relevance. It thereby provides an additional tool with which to further improve the understanding, correct diagnosis, and choice of therapy when treating patients with posterior shoulder instability.

The groups A, B, and C describe groups of PGHI with differing nature of pathology (first-time, dynamic, or static) and the subtypes further differentiate these groups in terms of their pathomechanism and provide a guideline in the choice of appropriate treatment. In general, the different subtypes can overlap or even co-exist (e. g., functional [B1] and structural [B2] dynamic PGHI). Additionally, the progression from one group or subtype to another is possible over time. A first-time dislocation (A2) might turn into a structural dynamic PGHI (B2) and if not adequately treated develop into an acquired static PGHI (C2) due to repetitive dislocations and progressive degenerative changes. Finally, the combined occurrence of AGHI and PGHI in terms of a bidirectional or multidirectional instability can also be observed in rare cases.

While a conservative treatment attempt is warranted in most patients with Type-1 PGHI including first-time posterior subluxation (A1), functional dynamic PGHI (B1), and constitutional static PGHI (C1), surgical treatment should be considered on an individual basis in patients with Type-2 PGHI including first-time posterior dislocation (A2), structural dynamic PGHI (B2), and acquired static PGHI (C2). Of course, the necessity of surgical treatment depends on the extent of the structural defects, severity of symptoms, chronicity, as well as patient-specific functional demand, age, and health status. Nonetheless, the ABC classification represents a guideline for the generally recommended type of treatment.

## Caption Electronic Supplementary Material

**Video 1:** Functional dynamic posterior instability (B1). Patient with functional posterior shoulder instability with aberrant muscle activation pattern.
